# Burden of typhoid fever and antimicrobial resistance in India (2023): a modelling study

**DOI:** 10.1016/j.lansea.2025.100714

**Published:** 2026-01-07

**Authors:** Vijayalaxmi V. Mogasale, Jacob John, Nikhil Sahai, Arindam Ray, Habib Hasan Farooqui, Vittal Mogasale, Bhim Gopal Dhoubhadel, W. John Edmunds, Andrew Clark, Kaja Abbas

**Affiliations:** a Department of Infectious Disease Epidemiology and Dynamics, London School of Hygiene & Tropical Medicine, London, UK; b School of Tropical Medicine and Global Health, Nagasaki University, Nagasaki, Japan; c Institute of Tropical Medicine, Nagasaki University, Nagasaki, Japan; d Department of Community Health, Christian Medical College, Vellore, India; e Department of Infectious Disease & Vaccine Delivery, Gates Foundation, New Delhi, India; f College of Medicine, Qatar University, Doha, Qatar; g Graduate School of Public Health, Yonsei University, Seoul, Republic of Korea; h Department of Health Services Research and Policy, London School of Hygiene & Tropical Medicine, London, UK; i Public Health Foundation of India, New Delhi, India; j National Institute of Infectious Diseases, Japan Institute for Health Security, Tokyo, Japan

**Keywords:** Typhoid fever, Incidence, Antimicrobial resistance, Disease burden, Fluoroquinolone-resistance, Vaccination, Typhoid conjugate vaccine, Prioritisation

## Abstract

**Background:**

India is one of the countries with a high typhoid fever burden. In 2022, the National Technical Advisory Group on Immunisation recommended including the typhoid conjugate vaccine (TCV) in the Universal Immunisation Programme. In this study, we aimed to estimate the 2023 burden of typhoid fever and its antimicrobial resistance (AMR) to inform targeted vaccine introduction strategies.

**Methods:**

We used a decision tree model to estimate typhoid cases, hospitalisations, complications, and deaths. Incidence and clinical parameters were derived from a multicentre Indian study, with state-wise AMR prevalence from a systematic review. Two co-primary and four alternative scenarios were presented to validate the robustness of the findings.

**Findings:**

We estimated 4.9 million (95% UI: 4.4–5.6) typhoid cases and 7850 (4300–14,900) deaths in India in 2023. Of 730,000 (534,000–970,000) hospitalisations, 600,000 (435,000–799,000; 82%) were attributable to fluoroquinolone-resistant. Under primary scenario A, children <5 years accounted for 321,000 (235,000–427,000; 44.0%) hospitalisations and 2600 (1300–4800; 34.0%) deaths. Under primary scenario B, 5–9 years of age accounted for 265,000 (135,000–278,000; 36.0%) hospitalisations and 2900 (1500–5300; 36.0%) deaths. Delhi, Maharashtra, and Karnataka together accounted for 29% of the national burden and had the highest rates of fluoroquinolone-resistant cases and deaths among the ten highest-burden states. Deaths linked to fluoroquinolone-resistance, multidrug resistance, third-generation cephalosporins, and azithromycin resistance were 4700 (1800–10,200), 122 (45–294), 183 (69–431), and 183 (68–432), respectively.

**Interpretation:**

Fluoroquinolone-resistance drives a large share of typhoid-related hospitalisations and deaths, especially in children under five and in high-burden states of India. Targeted TCV introduction, with broader age coverage among children, would maximise impact.

**Funding:**

WISE programme; 10.13039/501100023693Vaccine Impact Modelling Consortium; 10.13039/100009619Japan Agency for Medical Research and Development.

**Research in context**

**Evidence before this study**

We searched PubMed using search terms “Typhoid Fever” AND (“Incidence” OR “Burden”) AND “India” without language restrictions until July 31, 2025. We included peer-reviewed papers that presented data on typhoid fever incidence and mortality. We identified two data sources. The Surveillance for Enteric Fever in India (SEFI) study (2017–2020) collected primary data and reported site-to-site, urban-rural and age-specific variation in typhoid incidence in different Indian states. The second data source was the Global Burden of Disease (GBD) study 2021, which modelled a lower incidence but higher case fatality rate than the SEFI study. Then, we conducted a second PubMed search up to July 31, 2025, using the terms “Typhoid Fever” AND (Resistant∗) AND “India”, and selected relevant review articles. We also incorporated findings from our recently completed systematic review and meta-analysis of Salmonella Typhi AMR in India (1977–2024). Available data showed a persistently high fluoroquinolone resistance (>60%) steadily increasing from 1989 to 2024, peaking at 94% in 2017. Third-generation cephalosporin and azithromycin resistance remained consistently low, and multidrug resistance had steadily declined over the last three decades.

**Added value of this study**

This study aimed to estimate the typhoid burden by age, state, and antimicrobial resistance in India for 2023. We integrated typhoid fever incidence data from SEFI and state-specific AMR prevalence from our systematic review to estimate the typhoid burden in India in 2023, categorised by age, state, and AMR profile. We inferred a high impact of fluoroquinolone-resistance on hospitalisations and deaths, especially among children under five. Additionally, we identified priority states for vaccine introduction based on disease burden. We identified the typhoid fever mortality rate among treatment non-seekers and hospitalised cases with AMR-related complications as the major drivers of typhoid fever deaths. We evaluated these drivers under various scenarios to elucidate their implications for typhoid fever control activities, particularly vaccination.

**Implications of all the available evidence**

Our findings highlight the importance of targeting TCV in high-burden Indian states and prioritising children under five years of age. To achieve greater impact, broader catch-up or school-based vaccination campaigns should be implemented where feasible. We infer that AMR control is critical in reducing severe typhoid disease and mortality. We reiterate the value of typhoid conjugate vaccine (TCV) in AMR prevention and control alongside the broader strategy of antimicrobial stewardship and improvements in water, sanitation, and hygiene.

## Introduction

Typhoid fever, a systemic illness caused by *Salmonella enterica* serovar Typhi (*S*. Typhi), presents a significant health challenge in India.^1^ Despite several recent studies,^1^, ^2^, ^3^, ^4^ considerable uncertainty remains regarding the geographical and age-specific incidence and mortality of typhoid fever in India.

Surveillance for Enteric Fever in India (SEFI) is a population-based, multi-site study from 2017 to 2020 to estimate the incidence of typhoid fever among children aged 6 months to 14 years in four community-based sites and six hospital-based surveillance sites.^1^ The SEFI study found an incidence rate ranging from 35 to 1173 cases per 100,000 person-years, with 637 cases (95% confidence interval (CI): 567–713) per 100,000 person-years at community sites, and an incidence rate ranging from 12 (7–21) to 1622 (858–3359) cases per 100,000 person-years in hospital-based surveillance sites. Among individuals aged 15 and older, the incidence ranged from 108 (69–177) to 970 (683–1420) cases per 100,000 person-years in six hospital-based surveillance sites. Geospatial modelling of SEFI data showed state-wise incidence estimates from 149 to 1245 cases per 100,000 person-years, with a national mean incidence of 360 cases (297–494) per 100,000 person-years.^5^ This geospatial modelling study estimated 4.5 million (3.7–6.1) typhoid fever cases annually in India, with about 8930 (7360–12,260) deaths, based on a case fatality rate (CFR) of 0.2%.^5^ The Global Burden of Disease (GBD) study 2021 estimated an incidence rate of 263 (95% uncertainty interval (UI): 198–344) per 100,000 person-years in India, which is 27% lower than the GBD 2019 estimate of 315 (235–414).^3^^,^^6^ The GBD 2021 estimated 3.7 million (2.8–4.9) typhoid fever cases, 41,586 (20,815–68,514) deaths in India, based on a CFR of 1.1% (0.56–1.80).^6^ In GBD 2021, India contributed to 58% of global typhoid fever cases and 48% of global deaths.^3^

Antimicrobials are the primary treatment for typhoid fever, and the worldwide concern about antimicrobial resistance (AMR) is also profound in India.^7^, ^8^, ^9^ Before the 1990s, first-generation antimicrobials such as ampicillin, amoxicillin, chloramphenicol, and trimethoprim-sulfamethoxazole were commonly used. However, *S*. Typhi rapidly developed multidrug resistance (MDR) to these antibiotics.^10^ Currently, widely used antimicrobials include fluoroquinolones (such as ciprofloxacin or ofloxacin), cephalosporins (such as intravenous ceftriaxone or oral cefixime), and azithromycin. Resistance to these drugs has also become a significant challenge.^10^ A systematic review conducted in India revealed a 66% resistance rate to fluoroquinolones and less than 20% MDR between 2011 and 2015.^8^ In 2025, we conducted a systematic review and meta-analysis and tracked the changes from 1977 to 2024, focussing on the current AMR status of *S*. Typhi in India.^11^ We found nearly zero MDR, 63% resistance for fluoroquinolones and 3% each for third-generation cephalosporins and azithromycin after 2020.^11^ A 75-country modelling study estimated 94% fluoroquinolone-resistance and 9% MDR in India in 2019.^2^^,^^9^ A mathematical modelling study, which estimated the effect of vaccination on AMR-related typhoid fever in 73 countries, reported 37 million AMR typhoid fever cases and 366,000 deaths in India over 10 years from 2021. Of these, 21 million cases and 215,000 deaths were predicted to be averted by vaccination.^12^

Indian policymakers recommend using country-specific data to inform decision-making on typhoid fever control activities, such as the deployment of the typhoid conjugate vaccine (TCV) in the Universal Immunisation Programme (India's national immunisation programme). The 12th meeting of the National Technical Advisory Group on Immunisation (NTAGI) in India, held in 2016, has recommended enhanced collection of typhoid fever incidence data before further discussions and developing typhoid vaccine recommendations.^13^ This recommendation led to the establishment of the SEFI project in India from 2017 through 2020, which demonstrated a high incidence of typhoid fever, particularly in urban India.^1^ In 2022, upon the availability of India-specific data, the NTAGI recommended the introduction of the TCV in the Universal Immunisation Programme, primarily along with the measles-containing vaccine (MCV) at 9–12 months.^13^

In 2023, through evidence gap assessment and a key stakeholder survey, we identified research priorities to support further TCV decision-making in India.^14^ The health and economic burden of typhoid fever, including socio-economic burden, typhoid mortality, and the level of antimicrobial resistance was among the research priorities identified in our stakeholder survey. To address this, we developed a decision tree model integrated with updated data on typhoid fever incidence and clinical parameters to estimate the typhoid fever burden in India for 2023, categorised by age, state, and AMR levels.

## Methods

**Fig. 1:**
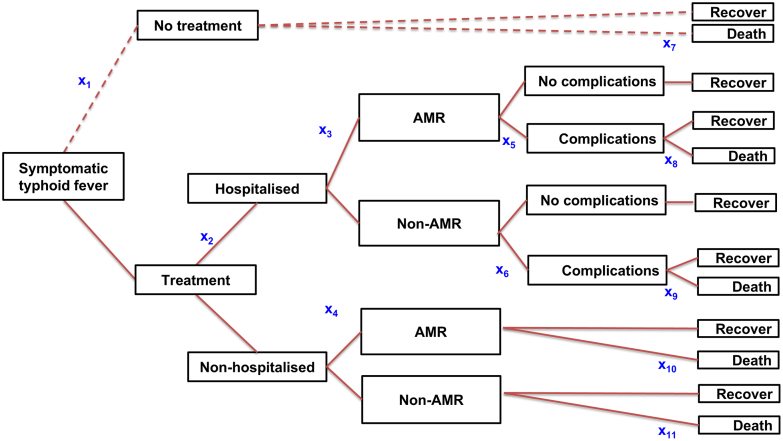
**Decision tree model for typhoid fever in India.** x_1_ to x_1__1_ represent the state transition probabilities. x_1_ is probability of no treatment among symptomatic typhoid fever cases; x_2_ is probability of hospitalisation among treated typhoid fever cases; x_3_ is probability of antimicrobials resistance (AMR) in hospitalised cases; x_4_ is probability of AMR in non-hospitalised cases; x_5_ is probability of complications in hospitalised AMR cases; x_6_ is probability of complications among hospitalised non-AMR cases; x_7_ is probability of death among non-treated symptomatic cases; x_8_ is probability of death among hospitalised AMR cases with complications; x_9_ is probability of death among hospitalised non-AMR cases with complications; x_10_ is probability of death among non-hospitalised AMR cases; x_11_ is probability of death among non-hospitalised non-AMR cases.

We developed a decision tree model to represent the epidemiology and clinical outcomes of typhoid fever in India for 2023, stratified by age, state, and AMR (Fig. 1). This model was initially developed for global-level analysis and validated by WHO expert reviews^15^^,^^16^ and adapted to the Indian context, incorporating additional recommendations from the WHO committee. The model was rooted with symptomatic typhoid fever cases and included branches for treatment, hospitalisation, AMR, typhoid fever-associated complications, and outcome (death or survival).

To assess the burden of AMR, we analysed four groups of antimicrobials.^11^ MDR was defined as concurrent resistance to ampicillin, chloramphenicol, and trimethoprim-sulfamethoxazole.^9^ We assessed fluoroquinolone resistance (FQR) by identifying the highest level of resistance to ciprofloxacin, ofloxacin, nalidixic acid, or pefloxacin within the same isolates. Third-generation cephalosporin resistance (3GCR) included resistance to ceftriaxone and other drugs in the same class. Azithromycin resistance (AZR) refers specifically to resistance against azithromycin. Population projections for 2023 (1.4 billion) were derived from the 2011 Census of India (Table 1).^17^ Age categories were organised by 6 months-4 years, 5–9 years, 10–14 years, and ≥15 years (Annex 1). We used state and union territory-specific typhoid fever incidence data from SEFI^1^^,^^5^ (Table 1). As the data lacked an age breakdown, we applied SEFI age distribution proportions from the primary sites^1^ to obtain age-stratified incidence by states (Annex 1 and 2).

**Table 1:** Input parameters, distribution, and data sources used in the typhoid fever decision tree model, along with the various scenarios used.

Parameter	Value	Type of distribution	Source	Comments
Population	1,388,163,000	–	^17^	Census of India projected data for 2023. State and age-wise population data are available in Annex 1
Incidence of typhoid fever (primary scenario)	Overall, 360 (297–494) per 100,000 PYs	gamma	^1^^,^^5^	Data from multi-site typhoid surveillance in India is organised by state and age group. Data is available in Annexes 1 & 2
Probability of incidence by age groups	Input by age-group	beta	^1^	Data from multi-site typhoid surveillance in India. Input by age-group: 6 months-4 years = 0.25 (0.20–0.33); 5–9 years = 0.36 (0.31–0.43); 10–14 years = 0.27 (0.22–0.33); ≥15 years 0.11 (0.08–0.16)
Probability of no treatment in symptomatic cases (x_1_)	0.038 (0.035–0.042)	beta	^1^^,^^18^	Healthcare utilisation survey conducted under SEFI India
Probability of hospitalisation (x_2_)	0.155 (0.118–0.204)	beta	^1^	Recalculated from SEFI data
Probability of AMR in hospitalised cases (x_3_)	0.908 (0.819–0.963)	beta	^11^	Estimated from the systematic review. Proportion of AMR in hospitalised cases versus non-hospitalised cases = 0.89. State-wise data is available in Annex 3
Probability of AMR in non-hospitalised cases (x_4_)	0.804 (0.725–0.853)	beta	^11^	Estimated from the systematic review. Proportion of AMR in hospitalised cases versus non-hospitalised cases = 0.89. State-wise data is available in Annex 3
Overall probability of FQR by Indian states	Ranged from 0.38 to 1	beta	^11^	Estimated from a systematic review. Each state had specific inputs. Logistic regression predicted FQR was used in scenario 2. See Annex 3. This was used to estimate x_3_ and x_4_
Probability of Multidrug Resistance (MDR)	0.02 (0.01–0.04)	beta	^11^	Estimated from a systematic review
Probability of third-generation cephalosporins resistance (3GCR)	0.03 (0.01–0.04)	beta	^11^	Estimated from a systematic review
Probability of azithromycin resistance (AZR)	0.03 (0.02–0.05)	beta	^11^	Estimated from a systematic review
Probability of complications in hospitalised AMR cases (x_5_)	FQR 0.040 (0.028–0.052)	beta	^1^	Recalculated from SEFI data
Odds ratio of complications in non-AMR versus AMR cases	0.496 (0.394–0.625)	lognormal	^19^	Literature review conducted by the WHO typhoid advisory group for the SAGE meeting
Probability of complications in hospitalised non-AMR cases (x_6_)	0.0197 (0.0196–0.0199)	beta	^1^^,^^19^	Calculated from x_5_ using the odds ratio of complications in non-AMR versus AMR cases
Probability of death (CFR) among symptomatic cases with no treatment (x_7_)	0.013 (0.006–0.020)	beta	^1^^,^^20^	Recalculated from SEFI data
Probability of death (CFR) among hospitalised AMR cases with complications (x_8_)	0.146 (0.038–0.255)	beta	^1^^,^^20^	Recalculated from SEFI data
Odds ratio of death in non-AMR versus AMR cases	0.850 (0.500–1.450)	lognormal	^19^	Literature review conducted by the WHO Typhoid Advisory Group for the SAGE meeting.
Probability of death (CFR) among hospitalised non-AMR cases with complications (x_9_)	0.124 (0.032–0.229)	beta	^1^^,^^19^	Calculated from ×8 using the odds ratio of deaths in non-AMR versus AMR cases
Probability of death (CFR) among non-hospitalised AMR cases (x_10_)	0.0005 (0.00–0.001)	beta	^1^^,^^20^	Estimated by SEFI data
Probability of death (CFR) among non-hospitalised non-AMR cases (x_11_)	0.0004 (0.00–0.0005)	beta	^1^^,^^19^	Calculated from ×10 using the odds ratio of deaths in non-AMR versus AMR cases
Probability of no treatment in symptomatic cases (x_1_) (alternative scenario 1)	0.149 (95% CI: 0.035–0.042)	beta	^1^^,^^18^	Healthcare utilisation survey conducted under SEFI India
Incidence of typhoid fever (alternative scenario 4)	Overall, 263 (198–344) per 100,000 PYs	gamma	^3^^,^^6^	IHME data by state and age group (available in Annex 2)
Probability of deaths (CFR) in typhoid fever cases (alternative scenario 4)	0.0112 (0.0056–0.0182)	beta	^3^	Based on the IHME typhoid fever GBD study 2021

Scenarios	Description
Primary scenario A	Uses decision tree input parameters derived from various publications of the Surveillance for Enteric Fever in India (SEFI) study,^1^^,^^5^^,^^18^^,^^20^ and our systematic review,^11^ and data from the literature review conducted by the WHO typhoid advisory group.^19^ Population data were obtained from the 2011 Census of India.^17^ The age distribution of hospitalisations, complications, and deaths was derived from the IHME typhoid fever GBD study 2021.^3^^,^^6^ It is also assumed that the four AMR groups (FQR, MDR, 3GCR, and AZR) overlap.
Primary scenario B	All input parameters remain the same as the primary scenario A, except we did not use age-group redistribution of hospitalisations, complications, and case-fatality rates. This scenario reflects the age distribution of typhoid fever cases and outcomes observed in the SEFI data.
Alternative scenario 1	All input parameters remain the same as in the primary scenario, except for the proportion of symptomatic cases with no treatment (x_1_), for which alternative assumptions from the SEFI study^1^^,^^18^ were used.
Alternative scenario 2	All input parameters remain the same as in the primary scenario, except that the prevalence of fluoroquinolone resistance was derived from a logistic regression model that predicted state- and union–territory–specific prevalence.
Alternative scenario 3	All input parameters remain the same as the primary scenario, except we assumed no overlap among the four AMR groups: FQR, MDR, 3GCR, and AZR. We summed the proportions of the four AMR types (capped at a maximum of 1).
Alternative scenario 4	All input parameters remain the same as the primary scenario, except typhoid fever incidence and case fatality rate (CFR), which were derived from the IHME typhoid fever GBD study 2021^3^^,^^6^

3GCR, third-generation cephalosporin resistance; AMR, antimicrobial resistance; AZR, azithromycin resistance; CFR, Case fatality rate; FQR, fluoroquinolone-resistance; GBD, Global Burden of Disease; IHME, The Institute for Health Metrics and Evaluation; PYs, person-years; SAGE, The Strategic Advisory Group of Experts on Immunisation; SEFI, Surveillance for Enteric Fever in India; MDR, multidrug resistance; WHO, World Health Organisation.

### Primary scenario: model parameters

We obtained the model parameters for the decision tree from the SEFI project, except for state-wise AMR data, which we derived from our systematic review (Table 1).^11^ Using the SEFI data, we calculated the required proportions for the decision tree model, applying the SEFI parameters uniformly across all age groups, states, and union territories. We derived the probability of symptomatic typhoid cases not seeking treatment (x_1_ in Fig. 1) from the SEFI community-based, multi-site healthcare utilisation survey, where 3.8% (409/10,664) of febrile illness cases were treatment non-seekers.^18^ We re-estimated the probability of hospitalisation (x_2_ in Fig. 1) to be 15.6% (45/290) based on the hospitalisation of blood culture-confirmed typhoid fever cases across four community-based SEFI surveillance sites.^1^^,^^20^

We used state and union territory-specific prevalence of FQR *S*. Typhi and national estimate of MDR, 3GCR, and AZR (x_3_ and x_4_ in Fig. 1) derived from our systematic review (Annex 3).^11^ The FQR data were available for 23 states and union territories. For 11 states that did not have their own data, we used meta-analysis-pooled prevalence data.^11^ Because other three groups of AMRs overlapped with the same case as FQR, their numbers were not added to FQR to avoid double-counting.

We utilised data from our systematic review to estimate the probability of FQR in hospitalised cases (x_3_ in Fig. 1) and non-hospitalised cases (x_4_ in Fig. 1).^11^ We compared the *S*. Typhi FQR prevalence in non-hospitalised (3276/4107) to the proportion of FQR in hospitalised cases (89/126) to estimate a ratio of 0.89 (FQR in non-hospitalised cases/FQR in hospitalised cases = 0.89). We then applied this ratio to state-wise *S*. Typhi FQR prevalence and SEFI hospitalisation rate (Table 1, Annex 3) to estimate the probability of FQR in hospitalised cases and non-hospitalised cases.

We recalculated the probability of complications in hospitalised FQR *S*. Typhi (41/1030 = 4%; x_5_ in Fig. 1) and the CFR among them (6/42 = 14.3%; x_8_ in Fig. 1) from SEFI data. The common complications of typhoid fever are intestinal perforation, gastrointestinal bleeding, shock, encephalopathy, myocarditis, hepatitis, cholecystitis and pneumonia. To estimate the probability of complications in hospitalised non-FQR cases (x_6_ in Fig. 1), we used data from a review conducted and presented by the typhoid expert committee in the WHO SAGE meeting,^19^ as SEFI had only 1% of non-FQR cases. The review presented odds ratios for four major complications in sensitive cases versus AMR cases: shock or hypotension (0.36), toxicity (0.50), gastrointestinal bleeding (0.36), and intestinal perforation (0.14), based on 19 studies reporting complications and deaths in 2311 AMR *S*. Typhi cases and 26 studies reporting complications and deaths in 3236 sensitive *S*. Typhi cases. We used the most conservative odds ratio of 0.50 (95% CI, 0.39–0.63) to estimate the probability of complication in hospitalised non-AMR cases (x_6_ in Fig. 1) compared to that in hospitalised AMR cases. Similarly, we used the odds of CFR (non-AMR versus AMR = 0.85; 95% CI 0.50–1.45) from the same review to estimate the probability of death (CFR) among hospitalised non-AMR cases with complications (x_9_ in Fig. 1) and non-hospitalised non-AMR cases (x_11_ in Fig. 1).^19^

We calculated the probability of death (CFR) among symptomatic cases with no treatment (x_7_ in Fig. 1) using four data points from SEFI: no treatment among symptomatic cases (3.8%) and no treatment before death (25/97 = 25.8%) from the health care utilisation survey,^18^ and estimated typhoid fever CFR among hospitalised (0.73%) and among non-hospitalised (0.05%; x_10_ in Fig. 1) typhoid fever cases.^20^ We obtained a CFR of 1.34% among symptomatic cases with no treatment (Table 1). We derived the CFR among hospitalised AMR cases with complications (6/41 = 14.6%; x_8_ in Fig. 1) from the SEFI data as well.^20^

The Surveillance for Enteric Fever in Asia Project (SEAP), conducted in Bangladesh, Nepal, and Pakistan, has demonstrated that typhoid fever hospitalisations varied by age group and were generally highest among those under 5 years of age.^21^ As age-group-wise hospitalisations could not be directly estimated from SEFI data, we used the fraction of total deaths for the respective age groups from the GBD 2021 study.^6^ We recalculated that 44%, 12%, 10%, and 34% of deaths occurred in the 6 months-4 years, 5–9 years, 10–14 years, and ≥15 years age groups, respectively, from the GBD 2021 study. We then applied these proportions to the respective age groups to redistribute hospitalisations, complications and deaths under primary scenario A. We removed the age-specific hospitalisation distribution derived from the GBD typhoid fever study under primary scenario B, directly reflecting the age distribution of typhoid fever incidence and its associated outcomes, as observed in the SEFI data.

### Alternative scenarios: model parameters

We conducted four alternative scenarios by changing our input parameter values (Table 1b).

In alternative scenario 1, we replaced the proportion of symptomatic cases with no treatment (x_1_ in Fig. 1) with 14.9%, combining a) febrile illness cases that did not seek treatment or practised self-medication (3.8%) and b) visited traditional healers or registered medical practitioners (informal term for people who practice medicine without formal qualifications) across six SEFI sites.^18^

In alternative scenario 2, we developed a logistic regression model to predict state- and union-territory–specific prevalence of FQR *S*. Typhi. Because the systematic review did not provide estimates for 11 states, the regression model was used to generate prevalence values, as presented in Annex 3.

In alternative scenario 3, we assumed no overlap among the four AMR categories: FQR, MDR, 3GCR, and AZR. In contrast to the primary scenario, which assumed complete overlap, this scenario treated each resistance type as mutually exclusive. We therefore summed the proportions of the four AMR groups, capping the total at 1.

In alternative scenario 4, we used state-wise typhoid fever incidence inputs from the GBD 2021 study, which are modelled estimates.^6^ The GBD study reported an overall typhoid fever CFR of 1.12% (95% CI: 0.57–1.80), and we recalibrated the proportional inputs in our decision tree model to fit the same overall CFR. The overall GBD incidence estimates for India were 37% lower than SEFI estimates, while the CFR was 6.2 times higher than SEFI estimates.

### Statistical tests used

We conducted a decision tree model analysis for each Indian state and union territory, totalling 34, categorised by age groups: 6 months-4 years, 5–9 years, 10–14 years, and ≥15 years for the year 2023. We conducted probabilistic and univariate sensitivity analysis using Monte Carlo simulation with 5000 iterations, implemented in Ersatz,^22^ a Microsoft Excel add-in program. Additionally, we carried out further analyses for MDR, 3GCR, and AZR by replacing the respective input values for AMR (Table 1) in the primary scenario. Then we ran scenario analysis. We estimated typhoid fever burden by age, state and AMR in India in 2023 and presented 1) typhoid fever cases by treatment, hospitalisation and deaths, 2) AMR typhoid fever cases by hospitalisations, complications, and deaths, 3) incidence rates of FQR *S*. Typhi cases and deaths, 4) non-AMR typhoid fever cases by hospitalisations, complications and deaths, 5) unknown AMR typhoid fever cases and deaths, 6) state- and age-specific cases by AMR status, hospitalisation, and deaths.

### Ethics statement

This study was approved by the London School of Hygiene & Tropical Medicine (LSHTM) Research Ethics Committee (Ref. No 31427, 27 November 2024).

### Role of the funding source

The funders had no role in study design, data collection, data analysis, data interpretation, or writing of the report. The authors had full access to all the data and final responsibility for the decision to submit for publication.

## Results

### Primary scenario analysis

#### Typhoid fever cases, hospitalisation and deaths

**Fig. 2:**
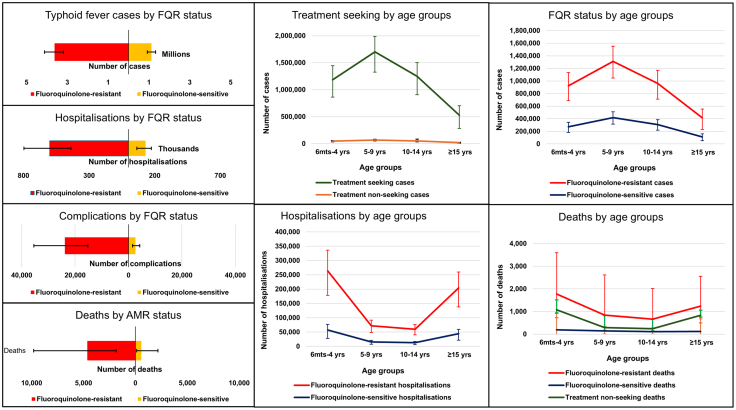
**Estimated number of typhoid fever cases, hospitalisations, complications, and deaths by fluoroquinolone-resistance status and age-groups in India for 2023 under primary scenario A.** Fluoroquinolone-resistant typhoid fever cases, hospitalisations, complications and deaths are relatively higher than fluoroquinolone-sensitive counterparts. Children aged 5–9 years exhibit the highest number of typhoid fever cases and AMR cases, while children aged 6 months to 4 years experience the highest number of hospitalisations and deaths. FQR, fluoroquinolone-resistance.

We estimated a median of 4.9 million (95% UI: 4.4–5.6) typhoid fever cases, 7850 (4300–14,900) deaths and 0.17% (0.01%–0.30%) CFR in India in 2023 (Table 2, Annex 4). Of these, approximately 4.7 million (4.2–5.3) symptomatic typhoid fever cases sought treatment, resulting in 0.73 million (0.53–0.97) hospitalisations, and 5300 (2000–12,100) deaths (Annex 4, Fig. 2). We estimated 2500 (1400–4100) deaths among symptomatic cases with no treatment and unknown AMR status (Fig. 2).

**Table 2:** Typhoid fever antimicrobial resistance burden in India in 2023.

Key parameters	Primary scenario A and B. Fluoroquinolone-resistance (FQR)	Alternative scenario 1 for FQR (Higher proportion of no treatment)	Alternative scenario 2 for FQR (Logistic regression predicted prevalence)	Alternative scenario 3 for AMR: No overlap (FQR+MDR+3GCR+AZR)	Alternative scenario 4 for FQR (GBD incidence and mortality data)	Multidrug resistance (MDR)	Third-generation cephalosporin resistance (3GCR)	Azithromycin- resistance (AZR)
	Median (95% UI)	Median (95% UI)	Median (95% UI)	Median (95% UI)	Median (95% UI)	Median (95% UI)	Median (95% UI)	Median (95% UI)
**Total typhoid fever**								
Typhoid fever cases: total	4,930,326 (4,386,695–5,546,000)	4,930,326 (4,386,695–5,546,000)	4,930,326 (4,386,695–5,546,000)	4,930,326 (4,386,695–5,546,000)	3,539,068 (3,114,394–4,001,654)	4,930,326 (4,386,695–5,546,000)	4,930,326 (4,386,695–5,546,000)	4,930,326 (4,386,695–5,546,000)
Typhoid fever symptomatic cases with treatment: total	4,741,996 (4,216,967–5,337,753)	4,201,544 (3,714,383–4,708,861)	4,741,996 (4,216,967–5,337,753)	4,741,996 (4,216,967–5,337,753)	3,403,715 (2,997,469–3,850,509)	4,741,996 (4,216,967–5,337,753)	4,741,996 (4,216,967–5,337,753)	4,741,996 (4,216,967–5,337,753)
Typhoid fever symptomatic cases with no treatment: total	188,706 (161,734–219,864)	734,998 (645,058–831,611)	188,706 (161,734–219,864)	188,706 (161,734–219,864)	135,510 (115,665–157,890)	188,706 (161,734–219,864)	188,706 (161,734–219,864)	188,706 (161,734–219,864)
Hospitalised typhoid fever cases: total	730,256 (533,690–970,022)	645,203 (475,906–859,752)	730,256 (533,690–970,022)	730,256 (533,690–970,022)	524,530 (387,437–700,181)	730,256 (533,690–970,022)	730,256 (533,690–970,022)	730,256 (533,690–970,022)
Non-hospitalised typhoid fever cases: total	4,004,683 (3,514,321–4,539,908)	3,551,318 (3,096,447–4,022,138)	4,004,683 (3,514,321–4,539,908)	4,004,683 (3,514,321–4,539,908)	2,877,324 (2,514,536–3,280,150)	4,004,683 (3,514,321–4,539,908)	4,004,683 (3,514,321–4,539,908)	4,004,683 (3,514,321–4,539,908)
Complications in hospitalised: total	26,174 (16,556–39,541)	23,105 (14,861–34,850)	26,174 (16,556–39,541)	26,174 (16,556–39,541)	18,419 (11,916–27,823)	26,174 (16,556–39,541)	26,174 (16,556–39,541)	26,174 (16,556–39,541)
Deaths with complications in hospitalised: total	3614 (1314–7821)	3206 (1158–6943)	3614 (1314–7821)	3614 (1314–7821)	12,729 (4755–27,973)	3614 (1314–7821)	3614 (1314–7821)	3614 (1314–7821)
Deaths in non-hospitalised: total	1243 (40–7127)	1113 (42–6149)	1243 (40–7127)	1243 (40–7127)	8092 (342–46,562)	1243 (40–7127)	1243 (40–7127)	1243 (40–7127)
Deaths in symptomatic cases with treatment: total	5272 (2028–12,135)	4653 (1812–10,605)	5272 (2028–12,135)	5272 (2028–12,135)	22,541 (7954–60,944)	5272 (2028–12,135)	5272 (2028–12,135)	5272 (2028–12,135)
Deaths in symptomatic cases with no treatment: total	2470 (1366–4112)	9445 (5204–16,058)	2470 (1366–4112)	2470 (1366–4112)	12,154 (5117–25,121)	2470 (1366–4112)	2470 (1366–4112)	2470 (1366–4112)
Deaths: total	7851 (4256–14,863)	14,371 (8789–22,642)	7851 (4256–14,863)	7851 (4256–14,863)	35,753 (17,558–75,509)	7851 (4256–14,863)	7851 (4256–14,863)	7851 (4256–14,863)

	Median (95% UI) (percentage)	Median (95% UI) (% of total)	Median (95% UI) (% of total	Median (95% UI) (% of total	Median (95% UI) (% of total)	Median (95% UI) (% of total)	Median (95% UI) (% of total)	Median (95% UI) (% of total)
**AMR typhoid fever**								
Typhoid fever cases: AMR	3,626,661 (3,189,790–4,114,025) (73.6%)	3,212,075 (2,814,489–3,622,217) (65.1%)	3,355,002 (2,942,844–3,817,172) (68.0%)	3,982,017 (3,492,410–4,487,896) (80.7%)	2,507,696 (2,169,094–2,896,241) (70.1%)	92,934 (61,449–144,256) (1.9%)	139,786 (98,360–203,170) (2.8%)	139,795 (98,548–202,338) (2.8%)
Hospitalised typhoid fever cases: AMR	598,225 (435,249–798,904) (81.9%)	528,025 (388,839–708,385) (81.8%)	570,172 (414,869–765,667) (77.9%)	633,274 (455,355–836,195) (86.7%)	414,090 (302,750–557,507) (79.0%)	15,868 (9655–26,194) (2.2%)	23,934 (14,994–37,401) (3.3%)	23,913 (15,031–37,167) (3.3%)
Non-hospitalised typhoid fever cases: AMR	3,020,428 (2,626,148–3,465,121) (75.4%)	2,679,566 (2,309,106–3,053,374) (75.5%)	2,786,367 (2,413,391–3,193,578) (69.5%)	3,342,871 (2,912,823–3,812,392) (83.3%)	2,091,978 (1,792,524–2,435,473) (72.7%)	76,918 (50,398–120,215) (1.9%)	115,575 (80,709–168,915) (2.9%)	115,604 (81,758–169,208) (2.9%)
Complications in hospitalised: AMR	23,622 (14,864–35,720) (90.3%)	20,810 (13,354–31,201) (90.1%)	22,327 (14,401–33,929) (87.9%)	24,905 (16,294–37,265) (93.0%)	16,273 (10,462–24,778) (88.4%)	628 (348–1126) (4.4%)	932 (546–1640) (6.5%)	949 (542–1619) (6.6%)
Deaths with complications in hospitalised: AMR	3307 (1208–7184) (91.5%)	2934 (1050–6323) (91.5%)	3082 (1171–6557) (89.5%)	3478 (1267–7609) (93.7%)	11,395 (4331–25,026) (89.5%)	87 (29–213) (5.4%)	131 (46–313) (8.2%)	133 (47–315) (8.0%)
Deaths in non-hospitalised: AMR	982 (32–5515) (79.0%)	873 (31–4780) (78.4%)	874 (28–4745) (72.8%)	1110 (38–5809) (85.9%)	6192 (261–34,590) (76.5%)	26 (1–143) (2.4%)	38 (2–209) (3.6%)	38 (2–213) (3.5%)
Deaths: AMR total	4652 (1803–10,214) (88.2%)	4082 (1610–8996) (28.4%)	4288 (1715–9142) (56.4%)	4977 (1925–10,815) (91.4%)	19,132 (6986–48,541) (53.5%)	122 (45–294) (2.2%)	183 (69–431) (3.3%)	183 (68–432) (3.3%)

	Median (95% UI)	Median (95% UI)	Median (95% UI)	Median (95% UI)	Median (95% UI)	Median (95% UI)	Median (95% UI)	Median (95% UI)
**Antimicrobial-sensitive**^a^**typhoid fever**								
Typhoid fever cases: non-AMR	1,120,181 (925,824–1,320,741)	989,536 (816,922–1,169,663)	1,380,020 (1,183,745–1,613,019)	765,596 (629,073–915,895)	892,436 (704,183–1,098,585)	4,648,535 (4,118,401–5,239,941)	4,611,455 (4,071,831–5178,319)	4,610,398 (4,058,290–5,179,538)
Hospitalised typhoid fever cases: non-AMR	130,748 (83,719–197,794)	114,753 (75,678–172,750)	160,897 (95,163–243,266)	95,546 (62,210–139,554)	109,632 (67,938–164,579)	718,365 (526,385–945,580)	706,874 (514,159–937,057)	708,992 (513,363–935,387)
Non-hospitalised typhoid fever: non-AMR	984,914 (820,880–1,165,869)	871,241 (729,168–1,029,301)	1,220,186 (1,037,456–1,430,146)	668,169 (553,776–796,297)	780,003 (620,557–961,837)	3,931,662 (3,443,205–4,458,038)	3,898,192 (3,409,774–4419,817)	3,896,488 (3,413,550–4,414,443)
Complications in hospitalised: non-AMR	2469 (1209–4857)	2186 (1096–4300)	3012 (1403–5911)	1814 (918–3525)	2073 (1017–4079)	13,593 (7179–25,377)	13,342 (7046–25315)	13,355 (7273–24,897)
Deaths with complications in hospitalised: non-AMR	275 (79–852)	245 (69–766)	331 (91–1082)	202 (56–626)	1160 (313–3724)	1523 (430–4679)	1460 (438–4609)	1520 (449–4412)
Deaths in non-hospitalised: non-AMR	254 (8–1704)	228 (8–1417)	312 (9–1933)	175 (7–1081)	1832 (73–11,749)	1079 (35–6664)	1026 (42–6261)	1050 (44–6502)
Deaths: non-AMR total	529 (86–2556)	473 (77–2183)	642 (100–3015)	376 (63–1708)	2992 (386–15,472)	2602 (465–11,343)	2486 (480–10869)	2570 (493–10,914)
**Unknown AMR typhoid fever**								
Total typhoid fever cases: unknown AMR	188,706 (161,734–219,864)	734,998 (645,058–831,611)	188,706 (161,734–219,864)	188,706 (161,734–219,864)	135,510 (115,665–157,890)	188,706 (161,734–219,864)	188,706 (161,734–219,864)	188,706 (161,734–219,864)
Deaths: unknown AMR typhoid fever	2470 (1366–4112)	9445 (5204–16,058)	2470 (1366–4112)	2470 (1366–4112)	12,154 (5117–25,121)	2470 (1366–4112)	2470 (1366–4112)	2470 (1366–4112)

Cases, hospitalisations, complications, and deaths due to antimicrobial resistance in four groups of antimicrobials used for treating typhoid fever.

AMR, antimicrobial resistance; MDR, Multidrug resistance; FQR, fluoroquinolone-resistance; 3GCR, third-generation cephalosporin resistance; AZR, azithromycin resistance; GBD, Global Burden of Disease.

a Antimicrobial sensitive refers to the antimicrobial listed at the top of the respective column. *S*. Typhi may be resistant to another antimicrobial.

#### Typhoid fever burden by age groups

In both primary scenarios, typhoid fever cases were highest among 5–9 years (1.8 million (1.5–2.2); 36%), followed by 10–14 years (1.3 million (1.0–1.6); 27%), and 6 months-4 years (1.2 million (1.0–1.6); 25%) (Fig. 2, Annex 4). Under primary sceanrio A, in total 320,000 (235,000–427,000; 44.0%) hospitalisations and 2000 (800–4100; 38.0%) deaths occurred in children aged 6 months-4 years, while 1000 (300–3400; 19.0%) deaths were among those aged 5–9 years and 800 (250–2600; 15.0%) deaths were among the 10–14year age group (Fig. 2, Annex 4). Among typhoid fever cases with treatment, <15-year-olds accounted for 4.2 million (89%) cases and 482,000 (66%) hospitalisations, 17,000 (66%) complications and 3900 (74%) deaths (Annex 4). Under primary scenario B, the highest rates of hospitalisations, complications, and deaths occured in children aged 5–9 years (36%), followed by those aged 10–14 years (27%) and under 5 years (25%). This results in a total of 88% of the burden affecting children under 15 (refer to Annex 3a–c).

#### Typhoid fever burden by FQR

3.6 million (3.2–4.1) typhoid fever cases, 0.59 million (0.44–0.79) hospitalisations and 4650 (1800–10,200) deaths were related to FQR (Table 2, Fig. 2, Annex 4). The FQR was 3.2, 4.6, 9.6, and 8.8 times more likely in typhoid fever cases, hospitalisations, complications and deaths, respectively, compared to fluoroquinolone-sensitive cases among treatment seekers (Fig. 2).

**Fig. 3:**
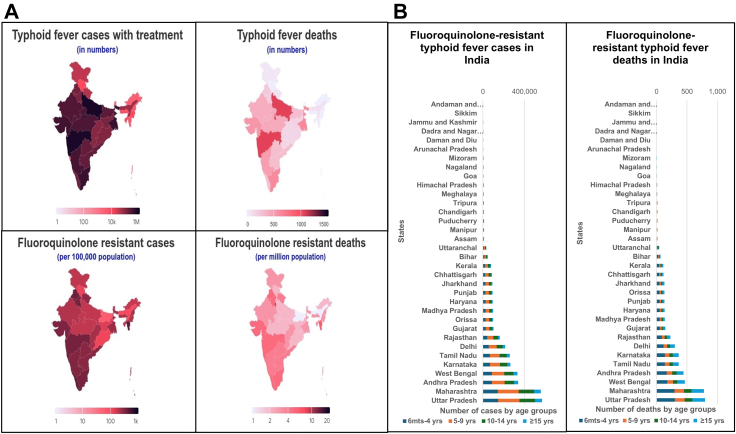
**Estimated burden of typhoid fever among the Indian states and union territories in 2023. a: Estimated number of typhoid fever cases and deaths and fluoroquinolone-resistant *S*. typhi incidence in India for 2023 by states and union territories.** Uttar Pradesh, Maharashtra, West Bengal, Tamil Nadu, and Andhra Pradesh (including Telangana) have the highest number of overall symptomatic typhoid fever cases and deaths, as shown in darker shades on the top two maps. Delhi, Chandigarh, Puducherry, Maharashtra, and Daman and Diu have the highest rates of fluoroquinolone-resistant typhoid fever incidence and mortality, as shown in darker colours on the bottom two maps. **b: Estimated fluoroquinolone-resistant typhoid fever cases and typhoid fever deaths for 2023 by age-groups in the Indian states and union territories under primary scenario A.** Uttar Pradesh, Maharashtra, West Bengal, Andhra Pradesh (including Telangana), Karnataka and Tamil Nadu have the highest number of fluoroquinolone-resistant typhoid fever cases across all age groups.

Uttar Pradesh, Maharashtra, West Bengal, Tamil Nadu, and Andhra Pradesh (including Telangana) accounted for 49% of typhoid fever cases and deaths (Fig. 3a). Delhi, Chandigarh, Puducherry, Maharashtra, and Daman and Diu were the top 5 states that had the highest rates of FQR *S*. Typhi incidence and mortality (Fig. 3b). Among the ten states with the largest number and highest incidence rates of FQR cases and deaths, three states, Delhi, Maharashtra, and Karnataka, contributed 29% of the overall FQR burden. We inferred through sensitivity analysis that the CFR in both hospitalised and non-hospitalised populations, as well as the probability of hospitalisation and complications, were the most sensitive input parameters driving overall deaths in the decision tree model (Annex 5a–c).

#### Typhoid fever burden by MDR, 3GCR and AZR

Analyses on MDR, 3GCR, and AZR showed 87 (29–213), 131 (46–313), and 133 (47–315) deaths, respectively, resulting from complications in hospitalised AMR cases (Table 2). Among non-hospitalised MDR, 3GCR, and AZR cases, the number of deaths was 26 (1–143), 38 (2–209), and 38 (2–213), respectively.

#### Alternative scenario analysis

In the alternative scenario 1 (higher proportion do not seek treatment), we estimated the number of typhoid fever cases to be the same at 4.9 million (4.4–5.6), 12% lower number of hospitalisations (645,000; 476,000–860,000), complications (20,800; 13,400–31,200) and deaths (2900; 1100–6300) among treatment seekers, while the deaths in typhoid fever cases with no treatment increased 6.9 times compared to the primary scenarios, resulting in overall deaths to be 14,400 (8800–22,600) (Table 2) and 0.30% (0.18%–0.45%) CFR.

In the alternative scenario 2 (logistic regression-based prediction of FQR), we estimated 272,000 (7.5%) fewer FQR cases (3.4 million; 2.9–3.8 million; 68.0%) and 364 fewer FQR deaths (4300; 1700–9100; 56.4%) compared to the primary analysis (Table 2). The top five states with the highest number of FQR cases remained unchanged from the primary scenario.

In the alternative scenario 3 (where four *S*. Typhi AMR groups were additive), the overall AMR cases increased by 9.8% (4.0 million; 3.5–4.5 million) and AMR deaths by 7.0% (5000; 1920–10,800) compared to the primary scenario (Table 2).

In the alternative scenario 4 (GBD incidence and CFR estimates), we estimated 28% lower typhoid fever cases and hospitalisations, and 31% lower AMR cases compared to the primary scenario. However, deaths in hospitalised cases, non-hospitalised cases, and overall increased by 3.5, 6.5, and 4.6 times, respectively, resulting in a total of 36,000 deaths (CFR = 1.1%; 0.5–2.1) (Table 2).

In the primary scenario, our estimates are close to SEFI's 4.5 million typhoid fever cases (3.7–6.1), 8930 deaths (7360–12,260), and 0.20% CFR.^5^ The alternative scenarios showed higher CFRs than the primary scenario, with alternative scenario 2 close to the GBD 2021 estimate of 3.7 million (2.8–4.9) cases, 41,586 (20,815–68,514) deaths, with a CFR of 1.1% (0.56%–1.8%).^6^

## Discussion

Our assessment of the typhoid fever burden in India by age, state, and AMR categories in 2023 has timely public health implications to inform targeted TCV introduction and prioritisation strategies in India. Our findings indicate that typhoid fever cases are highest among children aged 5–9 years, while hospitalisations and deaths are concentrated among children under 5 years in primary scenario A and among children aged 5–9 years in primary scenario B, reflecting alternative assumptions on age-specific hospitalisation patterns. Together with the high proportion of fluoroquinolone-resistant *Salmonella* Typhi infections among children under 15 years, these results inform age-based prioritisation for the introduction of TCV in India. We infer priority states for TCV introduction as Delhi, Maharashtra, and Karnataka, which have relatively the highest cases and FQR incidence rates. We infer that the disease and mortality burden associated with FQR is high, while the burden of other main groups of antimicrobials, namely MDR, 3GCR, and AZR, is low. Our scenarios reflected context-specific variability in typhoid fever CFR^23^ within India, influenced by factors such as socio-economic status, education, access, availability and perceptions of the quality of healthcare services.

We highlight the public health implications to inform the implementation of TCV in India. First, we estimated that the cases of typhoid fever and AMR are uniformly high across all children (5–9 years, followed by 6 months to 4 years, and 10–14 years), and hospitalisation and deaths are highest in <5-year-old or 5–9 years, depending upon the assumptions on hospitalisation age patterns. Therefore, TCV should be targeted to children <15 years through vaccination strategies such as catch-up campaigns or school-based initiatives, in addition to its introduction into routine immunisation programmes at 9–18 months. Relying solely on the routine immunisation programme, which targets TCV around 9 months, would be insufficient for the immediate control of typhoid fever in India, as it could take decades to reach older people, who also bear a significant disease burden. However, this decision should consider critical factors such as costs, budget impact, logistics, and operational feasibility. Second, we identified priority states for introducing the TCV and for planning and developing scale-up strategies. Among the top 10 states with the highest estimated number of typhoid fever cases and deaths, Delhi, Maharashtra, and Karnataka have the highest rates of FQR *S*. Typhi incidence and related mortality. These three states account for a quarter of the total typhoid fever cases and deaths in India, making them a priority for vaccine introduction based on disease burden criteria. Third, given the high AMR *S*. Typhi in India, the use of TCV could help address this challenge, as suggested in the WHO report, while making a case for vaccines in AMR control.^24^ However, we recognise that controlling AMR alone would not effectively reduce typhoid fever morbidity and mortality, indicating the need for comprehensive typhoid control measures, such as improving water and sanitation, food hygiene, and vaccination. Measures to control AMR are also essential, including antimicrobial stewardship programmes, infection prevention and control strategies, and enhanced monitoring of both AMR and antimicrobial usage.^24^^,^^25^ The TCV is anticipated to complement antimicrobial stewardship efforts by reducing unnecessary antibiotic prescriptions for non-specific febrile illnesses suspected to be typhoid fever.^26^ Fourth, we infer that sizable gains in invisible mortality can be achieved through the introduction of TCV. We estimated that around 2400 (1300–3900) deaths occur among treatment non-seekers. If TCV can reach these people, it could significantly reduce mortality.

India experiences a considerable burden of paratyphoid fever, a disease clinically similar to typhoid fever but caused by a different pathogen (*S*. Paratyphi) and is estimated to account for approximately one-third of the enteric fever burden.^1^ While TCVs do not prevent paratyphoid cases, they remain essential for controlling typhoid fever, and a combination vaccine targeting both pathogens (*S*. Typhi and *S*. Paratyphi) is under development.^27^

The high burden of FQR noted in our study has clinical practice implications. Empirical evidence suggests that clinicians have moved away from fluoroquinolones for treating typhoid fever and are increasingly using third-generation cephalosporins and azithromycin, based on observed clinical responses.^28^ The treatment practices across India need to further align with the changing patterns of AMR, based on evidence. The Government of India has published the most recent treatment guidelines in July 2025, excluding fluoroquinolones and recommending third-generation cephalosporins and azithromycin as the primary drugs.^29^ The guidelines also reinstated one of the first-line drugs, cotrimoxazole, as *S*. Typhi has regained susceptibility to it.^29^ However, there is a paucity of randomised clinical trials evaluating antimicrobial treatment regimens in India, the country with more than half of the global typhoid fever burden, which limits the evidence base for optimising treatment guidelines in the context of evolving AMR typhoid fever in India and beyond.^30^ Moreover, extensively drug-resistant (XDR) *S*. Typhi, resistant to first-line drugs, fluoroquinolones, and third-generation cephalosporins, has been reported in neighbouring countries (e.g. Pakistan), posing a significant risk of cross-border importation to India. Treatment options for XDR *S*. Typhi are limited, with azithromycin and carbapenems currently remaining the only effective therapies.^30^ Further research is needed to understand the clinical–laboratory correlation of azithromycin resistance in typhoid fever cases, as minimum inhibitory concentration (MIC) cut-off values do not consistently correlate with clinical response.

Our study has limitations. India is geographically complex and culturally diverse, with inequities in health access across the population. In our primary scenario, we represented the entire country using incidence and mortality data from a multi-site SEFI study.^1^ This was the feasible approach, as comprehensive data collection across all states and urban and rural areas was not possible. The morbidity and mortality data collected in active surveillance are likely to be underestimated because of improved access to health care during the surveillance. However, the mortality estimates among non-hospitalised cases and treatment non-seekers were predominantly derived from a large healthcare utilisation survey, which limits this bias.^18^ To gain a better understanding of potential underestimation, we also conducted alternative scenarios that represented higher mortality rates. We also note that, depending on assumptions regarding the age distribution of hospitalisations, the priority target age group may shift, although children under 15 years consistently remain the overall priority for vaccination. Ongoing laboratory-based typhoid surveillance established in urban India with support from WHO and Gavi is expected to provide clearer empirical evidence to further refine age-targeting strategies in the near term. Additionally, changes in AMR are dynamic and diverse across the healthcare system, making it challenging to quantify them across regions. We employed a systematic review and meta-analysis to derive the most recent (>2015) and state-representative data for AMR inputs. The systematic review of FQR indicated an apparent reversal of trends post-2019, which may not have been captured in our regression approach for predicting missing data. If resistance trends have indeed started to reverse, our estimates in the alternative scenarios could be overstated, even though the inputs for FQR are lower in the alternative scenarios than in our primary scenario.

In conclusion, our study demonstrated the ongoing burden of typhoid fever among children, its high prevalence in selected states, and a strong association with fluoroquinolone resistance. We emphasise targeted typhoid conjugate vaccination strategies for children and prioritise specific states for implementation and scale-up, highlighting their importance in supporting the health system's efforts to reduce mortality. The vaccination should complement antimicrobial stewardship and enhanced water, sanitation, and hygiene measures. We recommend conducting future research to understand the socio-economic burden of typhoid fever in India and to quantify the potential economic benefits of such investments.

## Contributors

Conceptualisation: VVM, WJE, AC, KA, VM; Data curation: VVM; Formal analysis: VVM; Funding acquisition: VVM, WJE, KA; Investigation: VVM; Methodology: VVM, WJE, AC, KA, VM; Project administration: VVM; Resources: VVM, JJ, NS, AR, HHF; Software: VVM;

Supervision: WJE, BGD, AC, KA; Validation: VVM, KA, VM; Visualisation: VVM; Writing—original draft: VVM; Writing—review & editing: JJ, NS, AR, HHF, VM, BGD, WJE, AC, KA; WJE, AC, and KA contributed equally. All authors have approved the final version. VVM, KA and VM have accessed and verified the data, and all authors were responsible for the decision to submit the manuscript.

## Data sharing statement

Data available within the article or its supplementary materials.

## Editor note

The Lancet Group takes a neutral position with respect to territorial claims in published maps and institutional affiliations.

## Declaration of interests

The authors declare no competing interests.

## Appendix A Supplementary data

Annex
