# Supplementary material for: Burden of typhoid fever and antimicrobial resistance in India (2023): a modelling study
Source: Lancet Reg Health Southeast Asia. 2026 Jan 7;44:100714. doi: 10.1016/j.lansea.2025.100714 (PMC12808886; doi:10.1016/j.lansea.2025.100714)
Supplement: Annex [file mmc1.docx]

## **Annex**

**Annex 1: State-wise population and typhoid fever incidence data by age-groups (primary scenario)**

|  | **State/union territory*** | **Total population (19)** | **Age categorised population 2023** | | | | **Typhoid incidence/100000 PYs Mean (95%UI)** | | | |
| --- | --- | --- | --- | --- | --- | --- | --- | --- | --- | --- |
|  |  |  | **6 m–4 y** | **5–9 y** | **10–14 y** | **≥15 y** | **6 m–4 y** | **5–9 y** | **10–14 y** | **≥15 y** |
| 1 | Andaman and Nicobar | 403,000 | 30,628 | 34,658 | 35,061 | 298,220 | 59 (38–105) | 85(89–140) | 62 (42–106) | 26 (16–52) |
| 2 | Andhra Pradesh | 91,246,000 | 6,934,696 | 7,847,156 | 7,938,402 | 67,522,040 | 99 (64–174) | 142 (99–230) | 105 (71–175) | 44 (26–87) |
| 3 | Arunachal Pradesh | 1,562,000 | 118,712 | 134,332 | 135,894 | 1,155,880 | 52 (35–93) | 74 (54–123) | 55 (88–93) | 23 (14–46) |
| 4 | Assam | 35,713,000 | 2,714,188 | 3,071,318 | 3,107,031 | 26,427,620 | 42 (29–76) | 61 (44–101) | 45 (32–77) | 19 (12–38) |
| 5 | Bihar | 126,756,000 | 9,633,456 | 10,901,016 | 11,027,772 | 93,799,440 | 41 (28–75) | 59 (43–99) | 43 (31–75) | 18 (11–37) |
| 6 | Chandigarh | 1,231,000 | 93,556 | 105,866 | 107,097 | 910,940 | 230 (143–400) | 330 (222–529) | 243 (158–401) | 102 (59–199) |
| 7 | Chhattisgarh | 30,180,000 | 2,293,680 | 2,595,480 | 2,625,660 | 22,333,200 | 77 (50–137) | 111 (78–181) | 82 (56–138) | 35 (21–68) |
| 8 | Dadra and Nagar Haveli | 699,000 | 53,124 | 60,114 | 60,813 | 517,260 | 113 (73–198) | 163 (114–261) | 120 (81–198) | 50 (30–98) |
| 9 | Daman and Diu | 564,000 | 42,864 | 48,504 | 49,068 | 417,360 | 143 (90–250) | 206 (141–331) | 151 (100–251) | 64 (37–124) |
| 10 | Delhi | 21,359,000 | 1,623,284 | 1,836,874 | 1,858,233 | 15,805,660 | 316 (191–554) | 454 (297–733) | 334 (212–556) | 141 (78–276) |
| 11 | Goa | 1,575,000 | 119,700 | 135,450 | 137,025 | 1,165,500 | 102 (67–176) | 146 (105–233) | 107 (75–177) | 45 (28–87) |
| 12 | Gujarat | 71,507,000 | 5,434,532 | 6,149,602 | 6,221,109 | 52,915,180 | 116 (74–203) | 167 (116–269) | 123 (82–204) | 52 (30–101) |
| 13 | Haryana | 30,209,000 | 2,295,884 | 2,597,974 | 2,628,183 | 22,354,660 | 100 (65–174) | 143 (101–231) | 105 (72–175) | 44 (27–87) |
| 14 | Himachal Pradesh | 7,468,000 | 567,568 | 642,248 | 649,716 | 5,526,320 | 38 (26–69) | 54 (40–92) | 40 (29–70) | 17 (11–35) |
| 15 | Jammu and Kashmir | 13,903,000 | 1,056,628 | 1,195,658 | 1,209,561 | 10,288,220 | 63 (42–113) | 91 (65–149) | 67 (46–113) | 28 (17–56) |
| 16 | Jharkhand | 39,466,000 | 2,999,416 | 3,394,076 | 3,433,542 | 29,204,840 | 76 (49–134) | 109 (77–177) | 80 (55–134) | 34 (20–67) |
| 17 | Karnataka | 67,692,000 | 5,144,592 | 5,821,512 | 5,889,204 | 50,092,080 | 112 (72–196) | 161 (112–259) | 118 (80–197) | 50 (29–98) |
| 18 | Kerala | 35,776,000 | 2,718,976 | 3,076,736 | 3,112,512 | 26,474,240 | 109 (71–189) | 156 (110–251) | 115 (78–190) | 49 (29–94) |
| 19 | Madhya Pradesh | 86,579,000 | 6,580,004 | 7,445,794 | 7,532,373 | 64,068,460 | 77 (51–136) | 111 (79–180) | 82 (56–137) | 35 (21–68) |
| 20 | Maharashtra | 126,385,000 | 9,605,260 | 10,869,110 | 10,995,495 | 93,524,900 | 131 (83–229) | 188 (129–303) | 138 (92–230) | 58 (34–114) |
| 21 | Manipur | 3,223,000 | 244,948 | 277,178 | 280,401 | 2,385,020 | 87 (56–153) | 125 (87–202) | 92 (62–154) | 39 (23–76) |
| 22 | Meghalaya | 3,349,000 | 254,524 | 288,014 | 291,363 | 2,478,260 | 64 (42–115) | 93 (65–152) | 68 (46–116) | 29 (17–57) |
| 23 | Mizoram | 1,238,000 | 94,088 | 106,468 | 107,706 | 916,120 | 113 (71–198) | 162 (111–262) | 119 (79–199) | 50 (29–99) |
| 24 | Nagaland | 2,233,000 | 169,708 | 192,038 | 194,271 | 1,652,420 | 67 (44–119) | 96 (69–157) | 71 (49–119) | 30 (18–59) |
| 25 | Odisha | 46,276,000 | 3,516,976 | 3,979,736 | 4,026,012 | 34,244,240 | 57 (38–102) | 82 (59–135) | 60 (42–102) | 25 (15–51) |
| 26 | Puducherry | 1,646,000 | 125,096 | 141,556 | 143,202 | 1,218,040 | 167 (103–294) | 240 (161–389) | 177 (115–295) | 75 (42–146) |
| 27 | Punjab | 30,730,000 | 2,335,480 | 2,642,780 | 2,673,510 | 22,740,200 | 108 (70–189) | 156 (109–250) | 114 (78–190) | 48 (29–94) |
| 28 | Rajasthan | 81,025,000 | 6,157,900 | 6,968,150 | 7,049,175 | 59,958,500 | 78 (51–138) | 112 (79–183) | 82 (56–139) | 35 (21–69) |
| 29 | Sikkim | 689,000 | 52,364 | 59,254 | 59,943 | 509,860 | 51 (35–90) | 73 (54–119) | 53 (38–90) | 23 (14–45) |
| 30 | Tamil Nadu | 76,860,000 | 5,841,360 | 6,609,960 | 6,686,820 | 56,876,400 | 125 (81–218) | 180 (126–288) | 132 (89–219) | 56 (33–108) |
| 31 | Tripura | 4,147,000 | 315,172 | 356,642 | 360,789 | 3,068,780 | 72 (47–128) | 104 (73–170) | 76 (52–129) | 32 (19–64) |
| 32 | Uttar Pradesh | 235,687,000 | 17,912,212 | 20,269,082 | 20,504,769 | 174,408,380 | 72 (47–127) | 103 (73–168) | 76 (52–127) | 32 (19–63) |
| 33 | Uttaranchal | 11,637,000 | 884,412 | 1,000,782 | 1,012,419 | 8,611,380 | 91 (60–159) | 131 (93–211) | 97 (66–160) | 41 (25–79) |
| 34 | West Bengal | 99,084,000 | 7,530,384 | 8,521,224 | 8,620,308 | 73,322,160 | 100 (64–147) | 144 (100–195) | 106 (71–148) | 45 (26–73) |
|  | **Total/average** | **1,388,163,000** | **10,55,00,388** | **11,93,82,018** | **12,07,70,181** | **1,02,72,40,620** | **91 (59–161)** | **131 (92**–**213)** | **97 (65–161)** | **41 (24–80** |

*We included data for 34 States and Union Territories; Telangana was included with Andhra Pradesh, Ladakh was included with Jammu and Kashmir, and Lakshadweep was excluded because there was no available incidence data for typhoid fever.

**Annex 2: Comparison of typhoid fever incidence by Indian states** **and union territories in two scenarios**

| **State/union territory*** | **SEFI estimates: typhoid fever incidence per 100,000 person years (primary scenario) Mean (95%UI)** | **GBD estimates 2021: typhoid fever incidence per 100,000 person years (alternative scenario Mean (95%UI)** |
| --- | --- | --- |
| Andhra Pradesh | 390 (321–534) | 224 (171–293) |
| Arunachal Pradesh | 204 (175–286) | 165 (124–218) |
| Assam | 166 (144–235) | 209 (157–275) |
| Bihar | 162 (140–230) | 225 (169–293) |
| Chhattisgarh | 305 (253–421) | 372 (280–490) |
| Delhi | 1245 (963–1702) | 226 (170–297) |
| Goa | 400 (339–540) | 11 (8–14) |
| Gujarat | 457 (374–624) | 171 (129–224) |
| Haryana | 393 (326–536) | 324 (244–426) |
| Himachal Pradesh | 149 (130–213) | 148 (113–194) |
| Jammu & Kashmir and Ladakh | 249 (210–346) | 75 (56–99) |
| Jharkhand | 298 (248–411) | 207 (156–270) |
| Karnataka | 441 (362–602) | 97 (73–127) |
| Kerala | 429 (356–582) | 7 (5–9) |
| Madhya Pradesh | 305 (255–419) | 466 (351–613) |
| Maharashtra | 515 (418–703) | 133 (100–175) |
| Manipur | 342 (283–470) | 280 (212–369) |
| Meghalaya | 254 (211–354) | 417 (312–548) |
| Mizoram | 444 (359–609) | 572 (428–755) |
| Nagaland | 264 (224–365) | 321 (240–421) |
| Odisha | 224 (190–313) | 231 (175–302) |
| Punjab | 427 (353–580) | 254 (193–333) |
| Rajasthan | 307 (256–424) | 526 (396–690) |
| Sikkim | 199 (174–276) | 281 (211–371) |
| Tamil Nadu | 494 (407–669) | 115 (87–151) |
| Tripura | 285 (237–394) | 107 (80–140) |
| Uttar Pradesh | 282 (235–390) | 451 (339–589) |
| Uttarakhand | 360 (302–490) | 369 (277–486) |
| West Bengal | 395 (323–453) | 74 (56–97) |
| **Average (India)** | **360 (297–494)** | **263 (198–344)** |

*We included data for 34 States and Union Territories; Telangana was included with Andhra Pradesh, Ladakh was included with Jammu and Kashmir, and Lakshadweep was excluded because there was no available incidence data for typhoid fever.

**Annex 3: Fluoroquinolone-resistance (FQR) for 2023 for Indian states and union territories based on systematic review and logistic regression modelling**

| **State** | **FQR prevalence estimated from the systematic review** | **FQR prevalence estimated in hospitalised (95%UI)** | **FQR prevalence estimated in non–hospitalised (95%UI)** | **Predicted overall FQR prevalence (95%UI)**** |
| --- | --- | --- | --- | --- |
| Andaman and Nicobar* | 0.93 (0.42–1.00) | 1.00 (0.95–1.00) | 0.94 (0.76–1.00) | 0.93 (0.70–1.00) |
| Andhra Pradesh | 0.97 (0.70–1.00) | 1.00 (0.99–1.00) | 1.00 (0.79–1.00) | 0.78 (0.64–0.92) |
| Arunachal Pradesh | 0.82 (0.74–0.87) | 0.91 (0.84–0.99) | 0.80 (0.67–0.96) | 0.86 (0.68–1.00) |
| Assam | 0.19 (0.06–0.45) | 0.21 (0.19–0.23) | 0.19 (0.16–0.22) | 0.66 (0.51–0.80) |
| Bihar | 0.24 (0.17–0.33) | 0.27 (0.25–0.29) | 0.24 (0.20–0.28) | 0.38 (0.04–0.73) |
| Chandigarh | 0.87 (0.67–0.96) | 0.96 (0.89–1.00) | 0.85 (0.71–1.00) | 0.89 (0.69–1.00) |
| Chhattisgarh | 0.99 (0.82–1.00) | 1.00 (1.00–1.00) | 1.00 (0.83–1.00) | 0.77 (0.64–0.91) |
| Dadra and Nagar Haveli | 0.82 (0.74–0.87) | 0.91 (0.84–0.99) | 0.80 (0.67–0.96) | 0.62 (0.45–0.78) |
| Daman and Diu | 0.82 (0.74–0.87) | 0.91 (0.84–0.99) | 0.80 (0.67–0.96) | 0.62 (0.45–0.78) |
| Delhi | 0.85 (0.68–0.94) | 0.94 (0.87–1.00) | 0.83 (0.70–1.00) | 0.83 (0.67–1.00) |
| Goa | 0.82 (0.74–0.87) | 0.91 (0.84–0.99) | 0.80 (0.67–0.96) | 0.93 (0.70–1.00) |
| Gujarat | 0.33 (0.23–0.45) | 0.37 (0.34–0.40) | 0.32 (0.27–0.39) | 0.73 (0.61–0.86) |
| Haryana | 0.84 (0.01–1.00) | 0.93 (0.86–1.00) | 0.82 (0.69–1.00) | 0.89 (0.69–1.00) |
| Himachal Pradesh* | 0.60 (0.52–0.67) | 0.66 (0.61–0.72) | 0.58 (0.49–0.70) | 0.84 (0.67–1.00) |
| Jammu and Kashmir* | 0.06 (0.00–0.50) | 0.06 (0.06–0.07) | 0.05 (0.05–0.07) | 0.76 (0.63–0.89) |
| Jharkhand | 0.82 (0.74–0.87) | 0.91 (0.84–0.99) | 0.80 (0.67–0.96) | 0.49 (0.23–0.75) |
| Karnataka | 0.92 (0.68–0.99) | 1.00 (0.94–1.00) | 0.92 (0.75–1.00) | 0.75 (0.62–0.87) |
| Kerala* | 0.54 (0.46–0.63) | 0.60 (0.56–0.65) | 0.53 (0.44–0.64) | 1.00 (0.72–1.00) |
| Madhya Pradesh | 0.39 (0.17–0.66) | 0.43 (0.40–0.47) | 0.38 (0.32–0.46) | 0.61 (0.44–0.78) |
| Maharashtra | 0.90 (0.82–0.95) | 1.00 (0.92–1.00) | 0.88 (0.74–1.00) | 0.71 (0.58–0.83) |
| Manipur* | 0.97 (0.66–1.00) | 1.00 (1.00–1.00) | 1.00 (0.79–1.00) | 0.60 (0.43–0.78) |
| Meghalaya | 0.82 (0.74–0.87) | 0.91 (0.84–0.99) | 0.80 (0.67–0.96) | 0.86 (0.68–1.00) |
| Mizoram | 0.82 (0.74–0.87) | 0.91 (0.84–0.99) | 0.80 (0.67–0.96) | 1.00 (0.71–1.00) |
| Nagaland | 0.82 (0.74–0.87) | 0.91 (0.84–0.99) | 0.80 (0.67–0.96) | 0.93 (0.70–1.00) |
| Odisha | 0.98 (0.79–1.00) | 1.00 (1.00–1.00) | 1.00 (0.81–1.00) | 0.54 (0.33–0.76) |
| Puducherry | 0.93 (0.42–1.00) | 1.00 (0.95–1.00) | 0.94 (0.76–1.00) | 0.89 (0.69–1.00) |
| Punjab | 0.75 (0.63–0.84) | 0.83 (0.77–0.90) | 0.74 (0.61–0.88) | 0.91 (0.69–1.00) |
| Rajasthan | 0.69 (0.37–0.89) | 0.76 (0.71–0.83) | 0.68 (0.56–0.81) | 0.69 (0.56–0.82) |
| Sikkim | 0.82 (0.74–0.87) | 0.91 (0.84–0.99) | 0.80 (0.67–0.96) | 0.92 (0.69–1.00) |
| Tamil Nadu* | 0.72 (0.69–0.75) | 0.24 (0.23–0.27) | 0.22 (0.18–0.26) | 0.71 (0.59–0.84) |
| Tripura | 0.82 (0.74–0.87) | 0.91 (0.84–0.99) | 0.80 (0.67–0.96) | 0.72 (0.60–0.85) |
| Uttar Pradesh* | 0.90 (0.81–0.95) | 1.00 (0.92–1.00) | 0.88 (0.74–1.00) | 0.66 (0.52–0.80) |
| Uttaranchal | 0.82 (0.74–0.87) | 0.91 (0.84–0.99) | 0.80 (0.67–0.96) | 0.80 (0.65–0.95) |
| West Bengal | 0.89 (0.72–0.96) | 0.99 (0.91–1.00) | 0.87 (0.73–1.00) | 0.65 (0.50–0.80) |
| **Pooled prevalence** | 0.82 (0.74–0.87) | 0.91 (0.84–0.99) | 0.80 (0.67–0.96) | **0.82 (0.74–0.87)** |

*The prevalence data from the systematic review represent meta-analysis results from 2015- 2024, except for states with * marks, where the data are prior to 2015. We used meta-analysis pooled prevalence data for 2015-2024 for states that did not have any FQR prevalence data.

**We used logistic regression to estimate the effect of nine explanatory variables on FQR prevalence for the remaining 11 states. The explanatory variables were mothers who had at least four antenatal care visits, total hospital beds per 100,000 population, the average mean surface air temperature from 1991–2020, the proportion of children under five who received antibiotic treatment for diarrhoea, the proportion of children under five who received antibiotic treatment for acute respiratory infections or fever, the proportion of children under five who sought treatment from health facilities for respiratory infections, the proportion of houses with improved sanitation facilities, the proportion of houses with access to improved water, and the prevalence of AMR pre–2015 as estimated in our systematic review. We first developed logistic regression models to assess the effect of all explanatory variables, but showed no significance in FQR prevalence due to collinearity issues. Subsequently, we examined the significance of each variable individually and found only one to be significantly associated with the outcome variable. The model indicated that for every 1% increase in the proportion of houses with improved sanitation facilities, the FQR prevalence rate increased by 0.01%, a statistically significant result. This variable explained approximately 16.7% (R-squared = 0.167, P value = 0.04) of the total variation in the current FQR prevalence. The predicted FQR prevalence with 95% confidence limits for 11 states and union territories with missing data for 2023.

**Annex 4: Estimated number of typhoid fever cases, hospitalisations, complications and deaths for the year 2023 in India by age and fluoroquinolone-resistance (FQR) status under primary scenario A and under primary scenario B**

| **4a. Primary scenario A: age distribution for hospitalisations, complications and deaths is applied from the global burden of disease study.** | | | | | |
| --- | --- | --- | --- | --- | --- |
| **Parameter** | **6m–4 y; Median (95% UI) (percentage)** | **5–9 y; Median (95% UI) (percentage)** | **10–14 y; Median (95% UI) (percentage)** | **≥15 y; Median (95% UI) (percentage)** | **Total; Median (95% UI) (percentage)** |
| **Total typhoid fever** |  |  |  |  |  |
| Typhoid fever cases: total | 1,246,041 (967,561–1,570,911) (25.3%) | 1,796,321 (1,479,591–2,159,600) (36.4%) | 1,318,510 (1,020,517–1,642,982) (26.7%) | 556,138 (363,483–797,600) (11.3%) | 4,930,326 (4,386,695–5,546,000) (100.0%) |
| Typhoid fever cases with treatment: total | 1,197,934 (931,385–1,510,549) (25.3%) | 1,727,617 (1,423,700–2,078,821) (36.4%) | 1,267,687 (981,900–1,580,166) (26.7%) | 534,761 (349,657–767,690) (11.3%) | 4,741,996 (4,216,967–5,337,753) (100.0%) |
| Typhoid fever cases with no treatment: total | 47,739 (36,280–61,347) (25.3%) | 68,689 (55,123–84,511) (36.4%) | 50,585 (38,633–64,469) (26.8%) | 21,282 (13,748–30,977) (11.3%) | 188,706 (161,734–219,864) (100.0%) |
| Hospitalised typhoid fever cases: total | 321,313 (234,823–426,810) (44.0%) | 87,631 (53,369–97,002) (12.0%) | 73,026 (53,369–97,002) (10.0%) | 248,287 (181,454–329,807) (34.0%) | 730,256 (533,690–970,022) (100.0%) |
| Non-hospitalised typhoid fever cases: total | 875,768 (608,874–1,178,522) (21.9%) | 1,639,588 (1,339,214–1,986,085) (40.9%) | 1,193,850 (912,110–1,504,791) (29.8%) | 284,209 (94,863–513,530) (7.1%) | 4,004,683 (3,514,321–4,539,908) (100.0%) |
| Complications in hospitalised: total | 11,516 (7,285–17,398) (44.0%) | 3,141 (1,987–4,745) (12.0%) | 2,617 (1,656–3,954) (10.0%) | 8,899 (5,629–13,444) (34.0%) | 26,174 (16,556–39,541) (100.0%) |
| Deaths with complications in hospitalised: total | 1,590 (578–3,441) (44.0%) | 434 (158–939) (12.0%) | 361 (131–782) (10.0%) | 1,229 (447–2,659) (34.0%) | 3,614 (1,314–7,821) (100.0%) |
| Deaths in non-hospitalised: total | 271 (9–1,605) (21.8%) | 504 (16–2,981) (40.5%) | 372 (12–2,148) (29.9%) | 83 (3–588) (6.7%) | 1,243 (40–7,127) (100.0%) |
| Deaths in cases with treatment: total | 1,994 (797–4,130) (37.8%) | 1,003 (320–3,409) (19.0%) | 780 (255–2,612) (14.8%) | 1,370 (540–2,838) (26.0%) | 5,272 (2,028–12,135) (100.0%) |
| Deaths in cases with no treatment: total | 622 (332–1,084) (25.2%) | 895 (489–1,518) (36.2%) | 658 (350–1,126) (26.6%) | 277 (137–515) (11.2%) | 2,470 (1,366–4,112) (100.0%) |
| Deaths: total | 2,644 (1,343–4,812) (33.7%) | 1,954 (1,051–4,475) (24.9%) | 1,482 (794–3,310) (18.9%) | 1,654 (802–3,146) (21.1%) | 7,851 (4,256–14,863) (100.0%) |
| **FQR typhoid fever** | **6m–4 y; Median (95% UI) (percentage)** | **5–9 y; Median (95% UI) (percentage)** | **10–14 y; Median (95% UI) (percentage)** | **≥15 y; Median (95% UI) (percentage)** | **Total; Median (95% UI) (percentage)** |
| Typhoid fever cases: FQR | 924,886 (717,674–1,169,706) (25.5%) | 1,307,131 (1,071,770–1,587,917) (36.0%) | 961,448 (741,065–1,207,264) (26.5%) | 418,687 (276,400–599,155) (11.5%) | 3,626,661 (3,189,790–4,114,025) (100.0%) |
| Hospitalised typhoid fever cases: FQR | 263,219 (191,510–351,518) (44.0%) | 71,787 (52,230–95,868) (12.0%) | 59,823 (43,525–79,890) (10.0%) | 203,397 (147,985–271,627) (34.0%) | 598,225 (435,249–798,904) (100.0%) |
| Non-hospitalised typhoid fever cases: FQR | 661,110 (456,133–896,034) (21.9%) | 1,235,393 (999,252–1,509,448) (40.9%) | 901,099 (684,962–1,142,994) (29.8%) | 213,976 (70,589–388,929) (7.1%) | 3,020,428 (2,626,148–3,465,121) (100.0%) |
| Complications in hospitalised: FQR | 10,394 (6,540–15,717) (44.0%) | 2,835 (1,784–4,286) (12.0%) | 2,362 (1,486–3,572) (10.0%) | 8,032 (5,054–12,145) (34.0%) | 23,622 (14,864–35,720) (100.0%) |
| Deaths with complications in hospitalised: FQR | 1,455 (532–3,161) (44.0%) | 397 (145–862) (12.0%) | 331 (121–718) (10.0%) | 1,124 (411–2,443) (34.0%) | 3,307 (1,208–7,184) (100.0%) |
| Deaths in non-hospitalised: FQR | 212 (7–1,264) (21.6%) | 399 (13–2,316) (40.6%) | 293 (10–1,674) (29.9%) | 64 (2–448) (6.6%) | 982 (32–5,515) (100.0%) |
| Deaths: FQR total | 1,775 (709–3,616) (38.2%) | 856 (283–2,728) (18.4%) | 667 (229–2,046) (14.3%) | 1,233 (488–2,555) (26.5%) | 4,652 (1,803–10,214) (100.0%) |
| **Fluoroquinolone-sensitive typhoid fever** | **6m–4 y; Median (95% UI) (percentage)** | **5–9 y; Median (95% UI) (percentage)** | **10–14 y; Median (95% UI) (percentage)** | **≥15 y; Median (95% UI) (percentage)** | **Total; Median (95% UI) (percentage)** |
| Typhoid fever cases: non-FQR | 274,899 (198,571–360,446) (24.5%) | 418,531 (327,586–524,422) (37.4%) | 305,823 (229,286–398,789) (27.3%) | 114,618 (66,576–175,089) (10.2%) | 1,120,181 (925,824–1,320,741) (100.0%) |
| Hospitalised typhoid fever cases: non-FQR | 57,529 (36,836–87,030) (44.0%) | 15,690 (10,046–23,735) (12.0%) | 13,075 (8,372–19,779) (10.0%) | 44,454 (28,465–67,250) (34.0%) | 130,748 (83,719–197,794) (100.0%) |
| Non-hospitalised typhoid fever cases: non-FQR | 215,896 (146,233–294,076) (21.9%) | 402,835 (314,612–506,284) (40.9%) | 292,892 (217,649–383,926) (29.7%) | 69,110 (22,707–127,856) (7.0%) | 984,914 (820,880–1,165,869) (100.0%) |
| Complications in hospitalised: non-FQR | 1,087 (532–2,137) (44.0%) | 296 (145–583) (12.0%) | 247 (121–486) (10.0%) | 840 (411–1,651) (34.0%) | 2,469 (1,209–4,857) (100.0%) |
| Deaths with complications in hospitalised: non-FQR | 121 (35–375) (44.0%) | 33 (9–102) (12.0%) | 28 (8–85) (10.0%) | 94 (27–290) (34.0%) | 275 (79–852) (100.0%) |
| Deaths in non-hospitalised: non-FQR | 56 (2–375) (21.9%) | 103 (3–685) (40.4%) | 74 (2–508) (29.3%) | 17 (0–134) (6.6%) | 254 (8–1,704) (100.0%) |
| Deaths: non-AMR total | 177 (36–750) (33.4%) | 136 (13–787) (25.6%) | 102 (10–593) (19.2%) | 110 (27–423) (20.8%) | 529 (86–2,556) (100.0%) |
| **Unknown FQR typhoid fever cases** | **6m–4 y; Median (95% UI) (percentage)** | **5–9 y; Median (95% UI) (percentage)** | **10–14 y; Median (95% UI) (percentage)** | **≥15 y; Median (95% UI) (percentage)** | **Total; Median (95% UI) (percentage)** |
| Total typhoid fever cases: unknown AMR | 47,739 (36,280–61,347) (25.3%) | 68,689 (55,123–84,511) (36.4%) | 50,585 (38,633–64,469) (26.8%) | 21,282 (13,748–30,977) (11.3%) | 188,706 (161,734–219,864) (100.0%) |
| Deaths: Unknown AMR typhoid fever | 622 (332–1,084) (25.2%) | 895 (489–1,518) (36.2%) | 658 (350–1,126) (26.6%) | 277 (137–515) (11.2%) | 2,470 (1,366–4,112) (100.0%) |
| **4b. Primary scenario B: No age distribution for hospitalisations, complications and deaths was applied** | | | | | |
| **Total typhoid fever** | **6m–4 y; Median (95% UI) (percentage)** | **5–9 y; Median (95% UI) (percentage)** | **10–14 y; Median (95% UI) (percentage)** | **≥15 y; Median (95% UI) (percentage)** | **Total; Median (95% UI) (percentage)** |
| Typhoid fever cases: total | 1,251,947 (963,904–1,576,446) (25.3%) | 1,794,395 (1,457,299–2,163,981) (36.3%) | 1,319,608 (1,034,876–1,642,903) (26.7%) | 552,811 (368,784–784,501) (11.2%) | 4,939,265 (4,339,948–5,566,344) (100.0%) |
| Typhoid fever cases with treatment: total | 1,203,963 (927,506–1,514,784) (25.4%) | 1,725,524 (1,402,773–2,078,699) (36.3%) | 1,269,726 (995,740–1,579,188) (26.7%) | 531,900 (354,445–754,650) (11.2%) | 4,748,426 (4,172,508–5,353,058) (100.0%) |
| Typhoid fever cases with no treatment: total | 48,040 (36,556–61,790) (25.4%) | 68,719 (54,576–85,147) (36.3%) | 50,605 (38,991–64,629) (26.7%) | 21,214 (13,878–30,582) (11.2%) | 189,233 (161,660–220,434) (100.0%) |
| Hospitalised typhoid fever cases: total | 185,227 (127,166–262,066) (25.4%) | 265,054 (134,733–273,977) (36.3%) | 194,700 (134,733–273,977) (26.7%) | 81,504 (50,049–127,886) (11.2%) | 729,953 (537,092–971,016) (100.0%) |
| Non-hospitalised typhoid fever cases: total | 1,016,116 (778,750–1,284,774) (25.4%) | 1,456,796 (1,180,264–1,772,245) (36.4%) | 1,071,048 (833,116–1,336,933) (26.7%) | 449,259 (298,724–637,783) (11.2%) | 4,006,107 (3,488,723–4,552,899) (100.0%) |
| Complications in hospitalised: total | 6,625 (4,046–10,376) (25.3%) | 9,512 (5,874–14,618) (36.4%) | 7,012 (4,323–10,913) (26.8%) | 2,917 (1,634–4,996) (11.2%) | 26,141 (16,727–39,450) (100.0%) |
| Deaths with complications in hospitalised: total | 908 (345–2,057) (25.3%) | 1,306 (490–2,842) (36.3%) | 957 (361–2,159) (26.6%) | 398 (147–959) (11.1%) | 3,593 (1,361–7,867) (100.0%) |
| Deaths in non-hospitalised: total | 322 (11–1,742) (25.0%) | 469 (16–2,490) (36.5%) | 343 (12–1,843) (26.7%) | 142 (5–799) (11.1%) | 1,285 (42–6,761) (100.0%) |
| Deaths in cases with treatment: total | 1,330 (517–3,092) (25.2%) | 1,917 (746–4,296) (36.3%) | 1,410 (547–3,165) (26.7%) | 587 (219–1,433) (11.1%) | 5,275 (2,076–11,713) (100.0%) |
| Deaths in cases with no treatment: total | 620 (327–1,061) (25.2%) | 890 (478–1,521) (36.2%) | 653 (349–1,143) (26.5%) | 271 (138–512) (11.0%) | 2,461 (1,348–4,066) (100.0%) |
| Deaths: total | 1,984 (1,046–3,820) (25.2%) | 2,857 (1,523–5,286) (36.3%) | 2,101 (1,116–3,920) (26.7%) | 873 (432–1,811) (11.1%) | 7,869 (4,248–14,437) (100.0%) |
| **FQR typhoid fever** | **6m–4 y; Median (95% UI) (percentage)** | **5–9 y; Median (95% UI) (percentage)** | **10–14 y; Median (95% UI) (percentage)** | **≥15 y; Median (95% UI) (percentage)** | **Total; Median (95% UI) (percentage)** |
| Typhoid fever cases: FQR | 919,877 (702,186–1,165,882) (25.4%) | 1,317,546 (1,063,347–1,604,116) (36.3%) | 970,174 (754,075–1,211,032) (26.7%) | 406,358 (270,046–578,957) (11.2%) | 3,627,569 (3,163,574–4,115,225) (100.0%) |
| Hospitalised typhoid fever cases: FQR | 152,132 (104,023–214,853) (25.4%) | 217,316 (154,423–303,035) (36.2%) | 159,747 (109,429–224,968) (26.6%) | 66,700 (40,712–105,183) (11.1%) | 599,821 (440,856–802,055) (100.0%) |
| Non-hospitalised typhoid fever cases: FQR | 765,095 (580,871–976,830) (25.3%) | 1,098,332 (880,250–1,348,565) (36.4%) | 807,510 (625,177–1,017,779) (26.7%) | 338,638 (223,778–484,368) (11.2%) | 3,019,281 (2,610,531–3,471,024) (100.0%) |
| Complications in hospitalised: FQR | 5,962 (3,648–9,406) (25.3%) | 8,582 (5,320–13,101) (36.4%) | 6,305 (3,860–9,844) (26.8%) | 2,621 (1,463–4,505) (11.1%) | 23,568 (15,055–35,496) (100.0%) |
| Deaths with complications in hospitalised: FQR | 832 (317–1,882) (25.4%) | 1,190 (443–2,579) (36.3%) | 876 (330–1,964) (26.7%) | 365 (134–869) (11.1%) | 3,280 (1,256–7,164) (100.0%) |
| Deaths in non-hospitalised: FQR | 252 (8–1,369) (25.1%) | 366 (13–1,946) (36.5%) | 269 (9–1,447) (26.7%) | 113 (4–620) (11.2%) | 1,005 (33–5,296) (100.0%) |
| Deaths: FQR total | 1,168 (460–2,617) (25.2%) | 1,683 (668–3,706) (36.3%) | 1,235 (481–2,680) (26.6%) | 514 (194–1,210) (11.1%) | 4,639 (1,864–9,976) (100.0%) |
| **Fluoroquinolone-sensitive typhoid fever** | **6m–4 y; Median (95% UI) (percentage)** | **5–9 y; Median (95% UI) (percentage)** | **10–14 y; Median (95% UI) (percentage)** | **≥15 y; Median (95% UI) (percentage)** | **Total; Median (95% UI) (percentage)** |
| Typhoid fever cases: non-FQR | 282,790 (210,743–367,900) (25.3%) | 406,407 (316,171–505,852) (36.3%) | 298,317 (225,279–385,404) (26.7%) | 124,994 (81,376–183,711) (11.2%) | 1,118,995 (920,014–1,316,246) (100.0%) |
| Hospitalised typhoid fever cases: non-FQR | 32,938 (20,125–52,353) (25.3%) | 47,357 (29,660–72,354) (36.4%) | 34,845 (21,552–55,113) (26.7%) | 14,638 (8,073–25,492) (11.2%) | 130,265 (83,794–195,981) (100.0%) |
| Non-hospitalised typhoid fever cases: non-FQR | 249,122 (185,695–324,577) (25.3%) | 357,456 (280,084–446,458) (36.3%) | 262,338 (198,953–339,538) (26.6%) | 109,799 (71,396–161,506) (11.1%) | 984,903 (817,085–1,159,400) (100.0%) |
| Complications in hospitalised: non-FQR | 627 (303–1,306) (25.3%) | 899 (436–1,829) (36.3%) | 663 (317–1,362) (26.8%) | 277 (125–616) (11.2%) | 2,478 (1,236–4,993) (100.0%) |
| Deaths with complications in hospitalised: non-FQR | 71 (20–228) (25.3%) | 102 (29–318) (36.5%) | 75 (21–237) (26.8%) | 31 (8–103) (11.1%) | 280 (80–882) (100.0%) |
| Deaths in non-hospitalised: non-FQR | 66 (2–417) (24.9%) | 96 (3–586) (36.3%) | 71 (2–425) (26.7%) | 30 (1–186) (11.1%) | 266 (8–1,586) (100.0%) |
| Deaths: non-AMR total | 137 (22–645) (25.1%) | 198 (32–904) (36.4%) | 146 (23–662) (26.7%) | 61 (9–289) (11.1%) | 545 (89–2,468) (100.0%) |
| **Unknown FQR typhoid fever cases** | **6m–4 y; Median (95% UI) (percentage)** | **5–9 y; Median (95% UI) (percentage)** | **10–14 y; Median (95% UI) (percentage)** | **≥15 y; Median (95% UI) (percentage)** | **Total; Median (95% UI) (percentage)** |
| Total typhoid fever cases: unknown AMR | 48,040 (36,556–61,790) (25.4%) | 68,719 (54,576–85,147) (36.3%) | 50,605 (38,991–64,629) (26.7%) | 21,214 (13,878–30,582) (11.2%) | 189,233 (161,660–220,434) (100.0%) |
| Deaths: Unknown AMR typhoid fever | 620 (327–1,061) (25.2%) | 890 (478–1,521) (36.2%) | 653 (349–1,143) (26.5%) | 271 (138–512) (11.0%) | 2,461 (1,348–4,066) (100.0%) |

*FQR= fluoroquinolone-resistance*

**Annex 4c: Comparison of estimated number of typhoid fever cases, hospitalisations, complications and deaths for the year 2023 in India by age and fluoroquinolone-resistance (FQR) status by primary scenario A versus primary scenario B.**

***** *Primary scenario A: Hospitalisations were redistributed age-group-wise based on the global burden of typhoid fever in the study of 2021. Primary scenario B: No age distribution for hospitalisations was applied. FQR= fluoroquinolone-resistance*

**Annex 5: Sensitivity analysis**

The Probabilistic Sensitivity Analysis conducted using Monte Carlo simulations identified the CFR in non-hospitalised cases, CFR in hospitalised cases with complications and FQR, CFR in typhoid fever cases with no treatment, proportion of complications in hospitalised FQR cases and proportion of hospitalisations as the top five sensitive parameters driving the uncertainty in overall deaths (Annex 5A). The univariate sensitivity analysis showed that the probability of complications, the probability of deaths in hospitalised FQR cases and the probability of hospitalisations were the top three input variables driving the overall deaths in the decision tree model (Annex 5B). The partial rank correlation coefficient indicated that the CFR in non-hospitalised cases and the CFR in hospitalised cases with complications were the top two most sensitive input variables driving overall deaths in the decision tree model. (Annex 5c).

**Annex 5a: Tornado graph showing Spearman’s rank correlation coefficient for the total number of typhoid fever deaths for 2023.** In multi-variate probabilistic sensitivity analysis, the case fatality rate (CFR) in non-hospitalised cases and the CFR in hospitalised cases with complications were the top two sensitive input variables driving the overall deaths in the decision tree model.

**Annex 5b: Tornado graph for the number of typhoid fever deaths for 2023.** In univariate sensitivity analysis, each input variable was changed by 10% from the mean, one at a time, in a Monte Carlo simulation to assess its effect on the overall number of typhoid fever deaths. The probabilities of complications and deaths in hospitalised fluoroquinolone-resistant cases were the top two input variables driving overall deaths in the decision tree model.


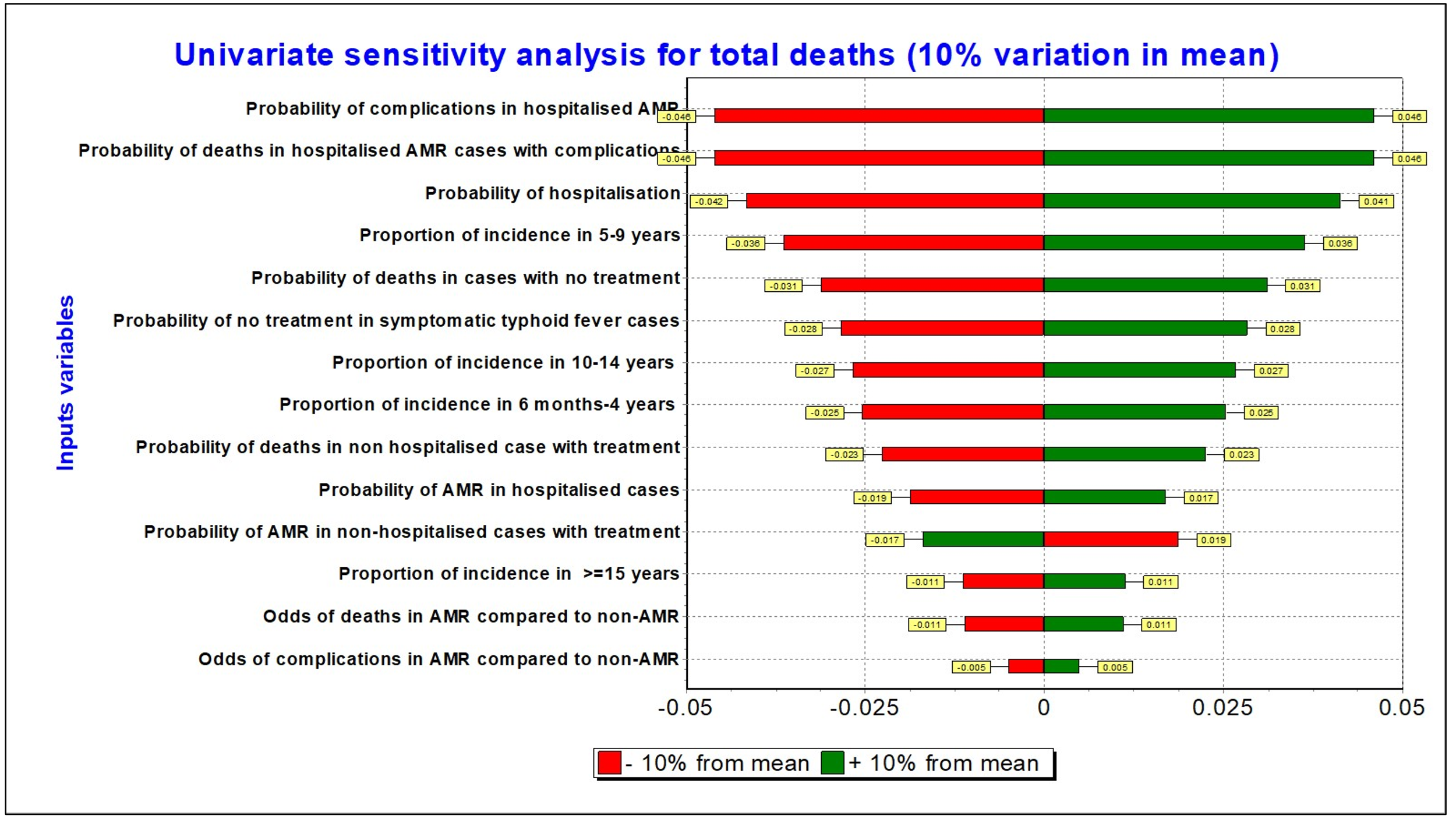


**Annex 5c: Tornado graph showing partial rank correlation coefficient for the total number of typhoid fever deaths for 2023.** The case fatality rate (CFR) in non-hospitalised cases and the CFR in hospitalised cases with complications were the top two sensitive input variables driving the overall deaths in the decision tree model.
